# Causal illusions in children when the outcome is frequent

**DOI:** 10.1371/journal.pone.0184707

**Published:** 2017-09-12

**Authors:** María Manuela Moreno-Fernández, Fernando Blanco, Helena Matute

**Affiliations:** Departamento de Fundamentos y Métodos de la Psicología, University of Deusto, Bilbao, Spain; Universidad de Granada, SPAIN

## Abstract

Causal illusions occur when people perceive a causal relation between two events that are actually unrelated. One factor that has been shown to promote these mistaken beliefs is the outcome probability. Thus, people tend to overestimate the strength of a causal relation when the potential consequence (i.e. the outcome) occurs with a high probability (*outcome-density bias*). Given that children and adults differ in several important features involved in causal judgment, including prior knowledge and basic cognitive skills, developmental studies can be considered an outstanding approach to detect and further explore the psychological processes and mechanisms underlying this bias. However, the outcome density bias has been mainly explored in adulthood, and no previous evidence for this bias has been reported in children. Thus, the purpose of this study was to extend outcome-density bias research to childhood. In two experiments, children between 6 and 8 years old were exposed to two similar setups, both showing a non-contingent relation between the potential cause and the outcome. These two scenarios differed only in the probability of the outcome, which could either be high or low. Children judged the relation between the two events to be stronger in the high probability of the outcome setting, revealing that, like adults, they develop causal illusions when the outcome is frequent.

## Introduction

Causal learning is an essential tool for adapting to the environment. Among other things, it allows for predicting changes, and for adjusting our behavior to deal with such changes. This ability is particularly relevant for children, given that they have limited experience with the world and, therefore, restricted knowledge about how it works. However, detecting causal relations is not an easy task, as it usually cannot be done directly. In fact, a crucial aspect that hinders causal inference is that sensory evidence does not explicitly include causal information. This forces people to infer causal relations indirectly by considering partial evidence from their senses. Moreover, sometimes these pieces of partial information are inconsistent with each other. For example, we would all agree that there is a relation between studying and passing an exam; however, this relation is far from being perfect and most students have the experience of failing an exam after studying, or scoring a good mark even if they did not prepare the contents at all. How, then, do people use this noisy evidence to infer that studying and passing an exam are actually related?

There are different indicators that can act as indirect cues for inferring causal links, and one of these is the contingency between the events. Contingency refers to the extent to which the potential cause and the alleged consequence co-vary (in the previous example, the extent to which studying and scoring a good mark correlate), and it is usually measured with the Δp index, computed as the difference between the probability of the consequence in the presence of the potential cause and in its absence [1,2]. A large number of studies have shown that humans are sensitive to the contingency between events [3–6], and there is also some evidence showing that even very young children may be able to infer causality by using co-variation patterns [7].

That children can learn about causality is not entirely surprising, given its relevance at this developmental stage. Children must learn how the world operates, gathering information to progressively build their knowledge. In this endeavor, they may explore, interact, and use their own intentional actions as instruments for learning; and they also need to be able to infer causal relations by other means such as observation [8,9].

Unfortunately, human abilities to detect causal relations are far from being perfect, and under specific circumstances, the judgments made by people regarding causal links may be easily biased. The resulting causal judgment can be so inaccurate that it may even lead to *causal illusions*, that is, people may perceive causal relationships between events that are actually unrelated [4,10,11]. Research on this field has revealed that some factors can systematically promote causal illusions (for a review see [12]). One of these factors is the outcome probability.

### Outcome probability and biased perceptions of causality

When evaluating the relation between a potential cause and its potential consequence, people typically overestimate the relation if the cause and the outcome are causally unrelated but the outcome occurs frequently [10,13–17]. This overestimation is usually known as the *outcome-density bias* or *outcome-frequency bias*. For example, imagine a supporter of a soccer team who watches all the matches on TV while sitting in the same seat because she believes that it could bring good luck and increase her team’s chances of winning. Clearly, the probability of winning is not enhanced by the enthusiastic fan watching the match while sitting in a “lucky armchair”, and the chances of winning would be the same regardless of whether the supporter is sitting in one armchair or another. However, if the team frequently plays well, the chances of winning are in any case high (outcome probability is generally high) and this may bias the fan’s perception about the causal link between both events, making her think that sitting in a specific place is actually effective. In fact, many sports fans exhibit similar superstitious behaviors and illusions of causality [18], some of which could be maintained by a high outcome probability.

On many occasions biased perceptions of causality may be innocuous, but they may also entail both positive and negative consequences, depending on the situation (see [19] for a recent review of this topic). For example, some superstitions could be regarded, to a certain extent, as adaptive behaviors because they may contribute towards promoting an unrealistic feeling of control (i.e. an *illusion of control* [20]) that may help in coping with the anxiety associated with the uncertain outcomes (e.g., taking an exam).

In this regard, deficits in perceived control have been related to anxiety [21] and obsessive compulsive disorders [22], and inducing unrealistic beliefs of control has proved to be an effective strategy for reducing the likelihood of experiencing a panic attack in patients with panic disorders [23]. Similarly, the illusions of control induced by the outcome-density effect have been negatively associated with dysphoric states and depressive symptomatology. Thus, depressed people have been shown to be less prone to the outcome-density effect, judging unrelated events more accurately than non-dysphoric controls (i.e., *depressive realism* [3,14,24]). In sum, there is evidence suggesting that some causal biases are linked to emotional well-being and self-enhancement. Unfortunately, not all causal misconceptions are positive or innocuous.

Misconceptions about causality can be extremely dangerous when they support hazardous behaviors. For example, in medical scenarios, the outcome-density bias can underlie the illusory belief that a bogus medical treatment is effective [25], even promoting resistance to accept a treatment that actually works if it is subsequently presented [26]. This situation may lead to very serious consequences in those cases in which the person is suffering from a lethal (but curable) disease, and the biased belief reduces chances of engaging in the effective therapy.

Positive and negative consequences of causal illusions have encouraged research on this topic, as well as the development of strategies aimed at controlling them. These strategies could be used either to encourage healthy illusions, or to prevent the harmful ones. For example, the depressive realism effect can be neutralized by encouraging people to process contextual information [27], and symptoms of laboratory-induced panic attacks can be reduced by inducing an illusion of control [22].

On the other hand, the negative consequences of cognitive biases have promoted interest in the issue of debiasing [28,29]. A variety of strategies have been proposed with the aim of reducing the negative effects of cognitive biases such as causal biases, by, for example, training people on normative rules, or by encouraging individuals to consider alternative hypotheses (for a review of some of these approaches see [30]). However, humans may exhibit some resistance to being debiased, as they have to be confronted with their own limitations for assessing reality [29,30].

Previous research has shown that people are less inclined to perceive their own biases than those of others, and that they are unwilling to admit or to perceive themselves as actually biased, even when they are confronted with evidence about this possibility. This asymmetric perception of bias is known as the *blind spot bias* [31,32] and it has been claimed to constitute a barrier to bias reduction [33]. Interestingly, the blind spot bias seems to have a developmental component, and, although children may also exhibit the bias, in some circumstances younger children are more willing than their older counterparts to admit that their own behavior is biased [34].

This willingness of children to acknowledge one’s biased performance could enhance the effect of strategies aimed at promoting positive bias and reducing negative bias, thus making childhood a more appropriate period for intervention than adulthood. Additionally, the instructional contexts in which children spend most of their time (i.e. schools or other educational settings) allow for controlled and standardized actions, facilitating the accessibility of intervention programs.

Nevertheless, before any intervention or developmental study is designed or tested, it becomes a priority to investigate whether those factors that encourage biased perception of causality in adults have any effect in children.

### Does outcome frequency affect causal judgments in children?

Research about the outcome-density bias has been primarily focused on adult performance. However, as we have already mentioned, some cognitive biases have a developmental component (e.g., blind spot bias), and it is also possible that the outcome-density effect displays changes depending on age. In fact, age differences have been reported in causal reasoning more generally. For example, when children are required to abstract an unusual causal principle, their reduced experience can make them more flexible and therefore more accurate than adults [35,36]; that is, they are more likely to contemplate infrequent or implausible causal hypotheses than adults. These unusual causal inferences and beliefs are sometimes called “magical thinking” [37], and they have been associated with children’s limited experience with the world [38].

There are reasons to believe that many instances of biases, including those that are causality-related, are a result of learning and previous experience with the world, rather than an innate tendency. This is the case, for example, of the widely known illusion of Muller-Lyer [39] in which two identical lines are perceived as different in length when they finish with inward or outward arrows. Recent research has pointed out the learned nature of this illusion [40], which has been explained as the result of a visual processing strategy based on the probability distributions of 3D-objects in the real world, that is, as a consequence of experience and acquired knowledge rather than an inborn strategy. The parallel between causal illusions and visual illusions (such as the Muller-Lyer) has been acknowledged in the past [12,19]; and it is possible that causal illusions—like visual illusions—also develop during our life as opposed to being an innate bias, which is another reason to investigate them. In line with these thoughts, research has shown that previous knowledge may act as a source of bias in causal assessment [41–45]. For example, contingency estimations and causal judgments can vary depending on prior assumptions about the causal role of the events (e.g., the stimuli as potential causes or as potential outcomes) [43].

Additionally, and apart from the contributions of previous knowledge, discrepancies between adults and children may also arise because of children’s own cognitive abilities and limitations. In the following paragraphs, we outline some factors that are involved to a certain extent in causal judgment, and that are likely to show variations depending on age, particularly when comparing children and adults. These factors may serve to further justify our interest in investigating outcome-density effects in children.

First, and to emulate real-world situations, experimental paradigms in conditioning and contingency/causal learning usually maintain some of the cognitive requirements of real-world experiences. Thus, in contingency learning tasks, the most widely used presentation format shows the information about the potential cause and the alleged consequence on a trial-by-trial basis [46–48]. More specifically, participants are sequentially presented with a series of events in which the cause and the consequence under assessment can be either present or absent. This presentation format requires the operation of working memory as an essential component for properly selecting, integrating, and combining all the information that is presented. Therefore, performance on this task becomes strongly dependent on working memory workload constraints. In fact, age differences in contingency learning have been reported with variations of the standard task, and related to memory changes throughout adulthood [49–52]. Thus, memory skills could also be a relevant factor in causal biases. It is also known that children and adults differ in their working memory abilities, with a considerable expansion in functional capacity throughout the early and middle school years to adolescence [53–56]. In conclusion, changes in working memory during childhood could affect causal learning, producing a different pattern of judgments in those situations where adults show outcome-density biases.

**Fig 1:**
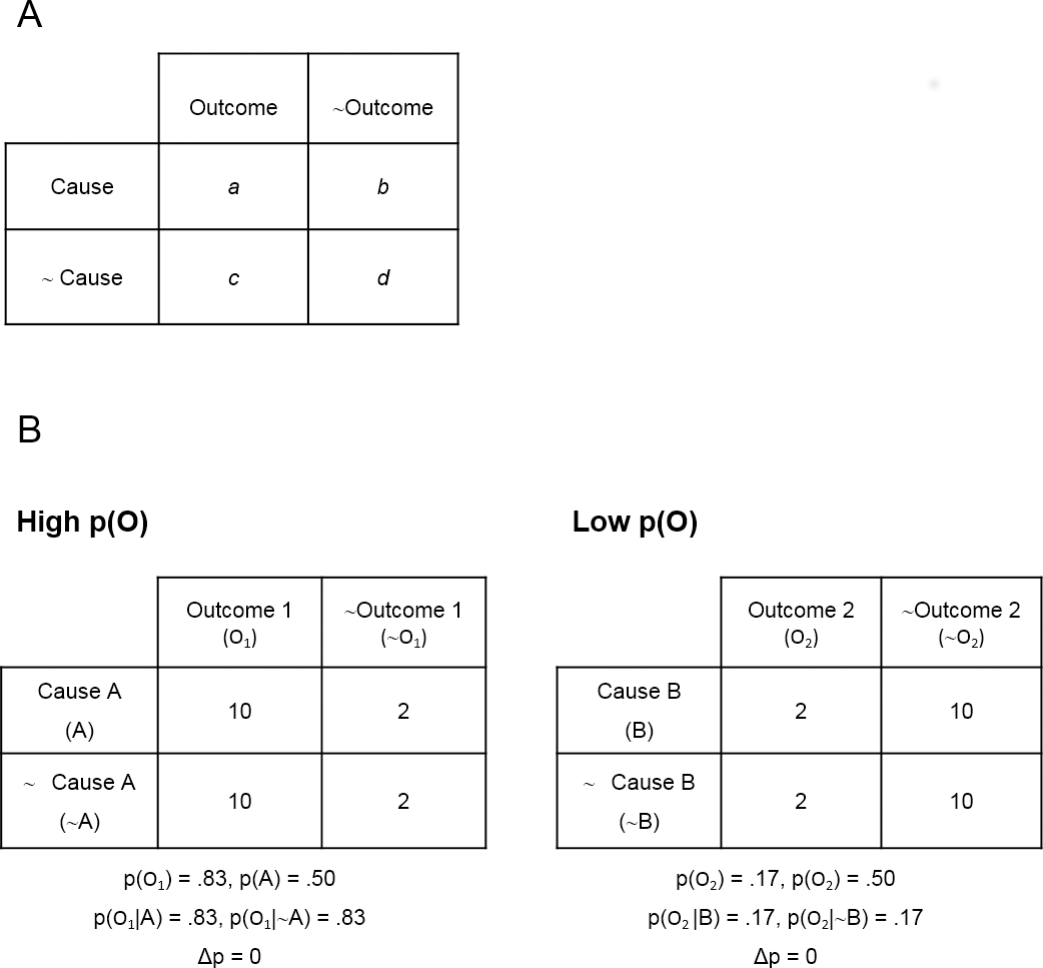
Contingency matrixes. Panel A shows the four possible cause–outcome combinations, and Panel B shows trial frequencies in the High and Low p(O) conditions of Experiments 1 and 2.

A second factor that could potentially lead to different patterns of causal biases for children when compared with adults is also related to operational abilities, more specifically, the ability to select and attend to certain types of information. In adults, one popular explanation for the outcome-density bias (and other causal biases) is an unequal weighting of the four types of information, presented in Fig 1 [57–60]. For example, when judging a causal relation, people usually consider as particularly meaningful and significant those events in which both the potential cause and the outcome co-occur, and as less relevant those events in which neither the cause nor the outcome occurs. However, a prerequisite for differentially weighting the events is being able to identify and discriminate between the different events shown in Fig 1. This ability would ultimately rely on perceptual, attentional, and operational components. Therefore, attentional control (i.e. attending to the specific cue and outcome under evaluation while filtering out irrelevant information) may be a crucial factor for causal learning and the development of causal biases. Again, these abilities are known to mature through childhood and early adolescence [61,62], suggesting that children might show different patterns from adults when judging causal relationships under outcome-density manipulations.

An additional factor that might be of interest is the ability of children to encode visual information. The literature on this topic suggests age differences in the tendency to process visual patterns (e.g., faces) as features or as configurations. In particular, it has been proposed that younger children tend to process and recognize visual patterns in an elemental way. In contrast, at ten years old, children perform as adults and tend to treat these stimuli as configurations [63–66], which has been known as “*the encoding switch hypothesis*” (see [67] for a recent review of this topic). Evidence about this encoding switch has been collected mostly in relation to face stimuli, although it may not be face-specific, appearing with other visual stimuli for which holistic processing can also be relevant [68–70]. Importantly, causal learning paradigms rely heavily on visual information and stimuli, and often make use of aggrupation or context-figure laws. Thus, the ability to encode and retrieve the individual features of visual scenes appears to be especially relevant for contingency learning in those situations in which the target stimuli (cue and outcome) must be pulled out from complex visual scenes that include additional elements (context). Although a deeper explanation will go beyond the scope of this report, it is worth noting that associative explanations of causal biases in adults attribute a significant role to contextual processing [27,71]. In sum, it seems reasonable to propose that children younger than ten years old may exhibit different processing of visual configurations that could affect their causal judgment under outcome-density manipulations.

As we have already mentioned, age-related differences in several cognitive processes (from perceptual to executive) are commonly reported in the literature. These differences, together with those related to knowledge about how the world works, may play a relevant role in contingency learning and in the development of causal biases. Hence, we argue that it is possible that children below ten years old do not show the same biases that are exhibited by adults. For this reason, before any comparative study (comparing performance of children against that of adults) is conducted, or interventions to modulate the bias designed, our first step must be to collect evidence for the actual existence of the outcome-density bias in children.

Some evidence already points towards the presence of an outcome-density bias before adulthood. For example, Barberia et al. [72] found that adolescents (*M*_*age*_ = 14 years old) develop causal illusions when the probability of the outcome is high. However, this evidence is not enough to ensure that the outcome density effect appears in children, not only because it refers to adolescents, but also because it may reflect the effects of additional factors beyond outcome density. Thus, in that experiment, the bias was partially mediated by the participants' behavioral tendency to expose themselves to a high number of cause-present trials, an additional factor that has also been shown to promote overestimations of causal relations on its own [73–76]. The procedure included the active intervention of participants who could choose if the potential cause was present or not, which added a strategic component beyond contingency learning. Consequently, and although these results may point towards an outcome-density bias before adulthood, further research is needed to confirm that the outcome density itself can induce causal illusions in children.

The present study will therefore examine the outcome-density effect in children younger than ten years old (before the alleged “perceptual encoding switch” and the complete maturation of the cognitive functions previously mentioned, but old enough to follow an adult-like procedure) by using a child-friendly version of the observational (or passive) contingency learning task [12] in which participants are simply exposed to the sequence of events, without deciding themselves when the cause was to be present. Although active procedures are a common and suitable option to assess the outcome density effect, we chose to use the observational procedure to avoid differences in cue density derived from the active intervention of participants, and also to hinder any attributions about self-performance that may complicate the evaluation of the outcome-density as a source of bias by itself.

## Experiment 1

Experiment 1 was designed with the main goal of testing whether the high probability of the outcome encourages causal illusions in children in the same way as in adults by comparing, in a within-subjects design, two conditions varying only in outcome-density (High vs. Low). Although between-subjects designs are a common approach to test the outcome density effect in adults [15,75], within-subjects designs can also be a suitable alternative [13,77], and they offer an additional advantage for testing the effect in children: They provide a reduced error variance associated with individual differences, which might be important in this population. As we have outline previously, some cognitive abilities that can be relevant for causal inference are still developing during childhood, and they may noticeably differ between children within the same age range. This makes a counterbalanced within-subjects design the best approach to control for the potential effects of developmental differences.

To promote internal validity as much as possible, Experiment 1 employed an adaptation of the observational procedure used by Matute et al. [78], that we modified for use with children. Thus, we maintained the videogame format to make the task attractive, but removed all written texts so that the children were not required to read at any point (instructions were provided verbally by the experimenter). The cover story was also modified, and the original medical scenario changed to make it comprehensible and child-friendly. Finally, we did not ask children for predictions about the outcome (as in the original version of the task) to avoid them feeling uncomfortable if they failed in their predictions, and to encourage them to focus only on the relation between the cue and the outcome, rather than on their own performance.

Participants were asked to play the role of a judge in a planting contest, and to assess the ability of two different characters to make plants grow. Each of these two characters was presented as participating in the contest on several occasions; on some occasions, he was required to plant but on others he was not. In this way, children had the opportunity to witness if the plants grew or not, either when the characters had planted or when they had not. The children saw the two characters perform sequentially (one after the other), after which they were required to award a trophy to the best farmer. Both characters were actually poor farmers, as the contingency between planting and the plants growing was set to zero in both conditions. However, the plants grew with a high probability for one character and with a low probability for the other. That is, the outcome probability was manipulated to promote an outcome-density effect that is expected to bias children’s causal judgments.

### Materials and methods

#### Ethics statement

The ethical review board of the University of Deusto examined and approved the procedure used in this experiment (Ref: ETK-12/14-15).

#### Participants

Twenty-eight children (22 girls) ranging from 7 to 8 years old (87 to 102 months, *M* = 94.04, *SD* = 3.95) were recruited from a public school of a rural town. The school was initially provided with a detailed explanation about the goals of the research, methods, and data treatment. Further details were provided in a personal meeting, and written consent forms were submitted to the parents/legal guardians through the school.

Only those children whose parents gave prior signed consent were offered the opportunity to take part in the study, and the verbal agreement of each child was required to participate (all children agreed to take part in the study).

#### Stimuli and apparatus

The experiment was presented as a web application based on World Wide Web Consortium (W3C) standards (i.e., HTML, CSS, and JavaScript) displayed full screen on a 15.4-inch laptop. The program is available at the Open Science Framework [79].

**Fig 2:**
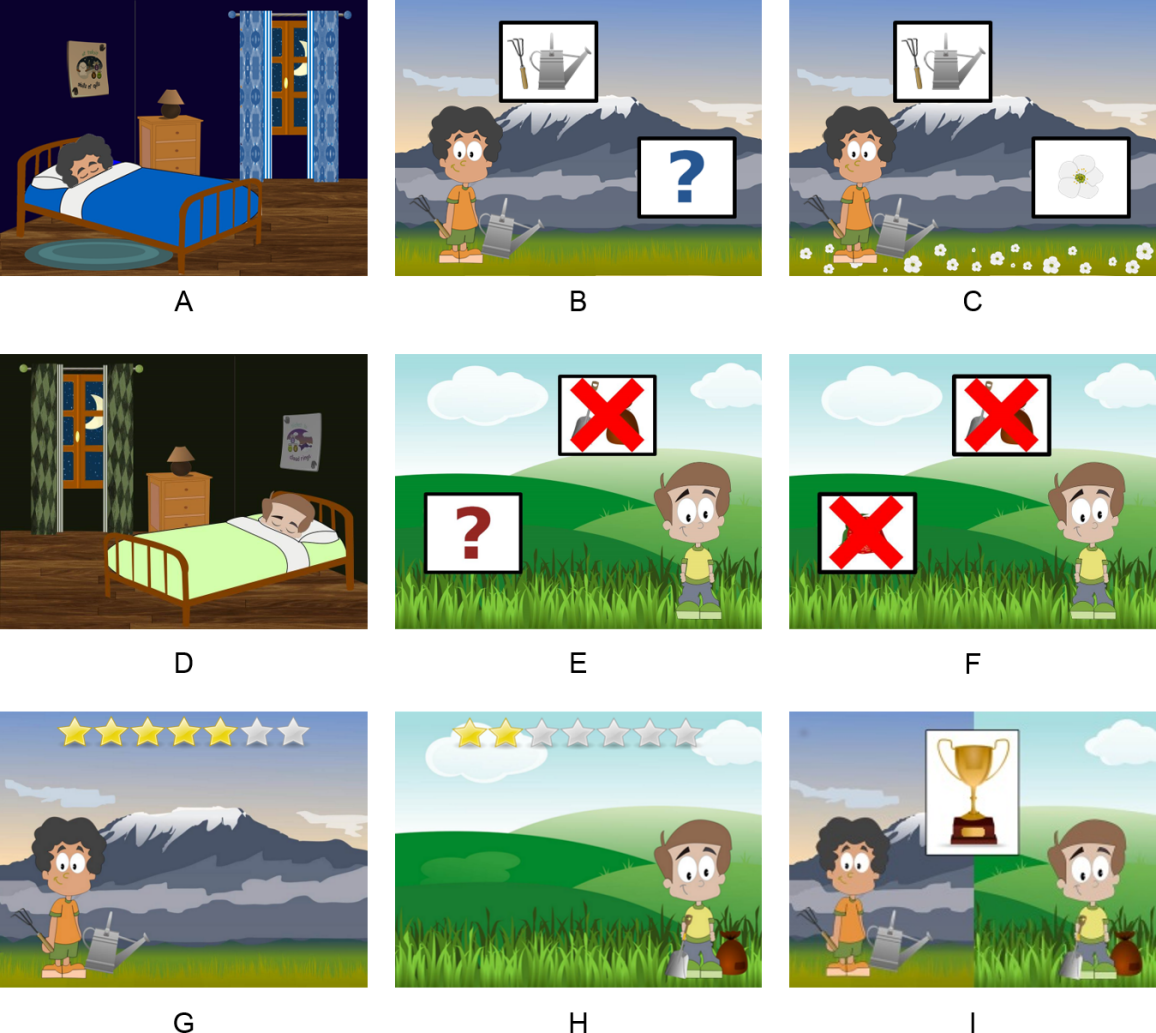
Example of images used in Experiment 1. The first and second rows depict the training trial structure for each storyline. The panels correspond to: Inter-trial interval screens (panels A and D), Cue screens (panels B and E), and Outcome screens (panels C and F). Contestants carried tools only on cue-present trials (the cue-box placed on the upper side of the screen appeared crossed out otherwise). On the outcome screens, plants covered the ground on outcome-present trials (the outcome-box appeared crossed out otherwise). The third row depicts the individual evaluation screens (panels G and H), and the final trophy assignment screen (panel I). The figures were adapted from images retrieved from https://openclipart.org under a CC BY license, with permission from https://openclipart.org, original copyright CC0.

We created two scenarios for the planting contest by using copyright-free images. Each scenario was associated with one of two characters, and they differed in layout (left or right oriented), contest background (mountainous or country), gardening tools (rake and watering can, or seed bag and shovel), and plants (flowers or strawberries). For each scenario, we also created an additional image to be used during the inter-trial intervals. These images represented the characters while they were sleeping during the night before each day of the contest. For examples, see Fig 2.

#### Procedure and design

**Fig 3:**
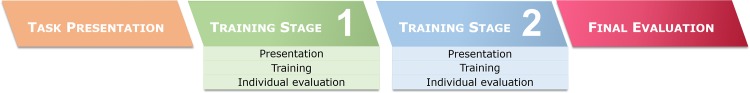
Procedure flow chart. The two scenarios (character, plants, layout, contest background, gardening tools, etc.) and the probability of the outcome were assigned to Training Stage 1 and Training Stage 2 counterbalanced across participants.

The children were brought to a room in their school and individually tested in a single session lasting approximately fifteen minutes. The participants sat next to the experimenter with a laptop positioned on a low table in front of them. After collecting their gender and age, the experimenter verbally presented the game (a procedure flow chart is presented in Fig 3).

The participants were told that they were going to watch two characters, one planting strawberries and the other planting flowers, and that they would have to observe their performance in order to award the trophy to the best farmer. After these initial instructions, a starting screen with “Contestant 1” written on top was presented and the first stage announced (see S1 Appendix for additional details). Throughout this stage, participants sequentially observed 24 trials related to the first contestant. Each trial started with an inter-trial interval in which one of the two characters appeared in his bedroom while sleeping (see Fig 2A and 2D). The child was asked to wake him up by clicking with the mouse, as the experimenter said: “Let’s wake him up so that he can go to the contest!” Immediately after clicking, a new screen with the contestant in the countryside was presented (see Fig 2B and 2E).

There were four types of trials, corresponding to the four cells in the matrix in Fig 1A (i.e. they presented a combination of the presence/absence of cause and outcome). In this task, the potential cause of plants growing was the contestants' attempt to plant, and the outcome was plants growing or not. On some trials (cause-present trials), the contestant attempted to plant the seeds, while on other trials (cause-absent trials), he did not, and just remained watching. This information was conveyed visually. Thus, on “a” and “b” trials, the character carried his gardening tools with him, whereas on “c” and “d” trials, he did not have the tools. This information was highlighted by including a box on the screen (i.e., "cue-box") that contained either the gardening tools (type “a” and “b” trials), or the gardening tools crossed out in red (type “c” and “d” trials), as shown in Fig 2B and 2E. Furthermore, the experimenter pointed to the cue-box on each trial stressing its status verbally: “Look, he is [is not] planting today. He is [is not] carrying the tools”.

A similar box but with an interrogation mark appeared at the bottom of the picture (outcome-box). The participants were asked to click on this box to proceed to the outcome screen, and to discover whether or not the plants had grown. A plant (either crossed out or not) then appeared instead of the question mark, and the ground was covered with plants (in outcome-present trials) or stayed without changes (in outcome-absent trials) as displayed in Fig 2C and 2F. Outcome presence or absence was also verbally stressed on each trial: “Look, flowers [strawberries] have [have not] grown, let’s see what will happen on the next day”.

After 24 training trials, the participants observed the individual evaluation screen (see Fig 2G and 2H) and were asked to rate the contestant by giving him stars ranging from 0 (very bad performance) to 7 (very good performance). In adults, cue-outcome relations are usually assessed by using numerical ratings (for example, using scales between 0 and 100) [14,26], but young children are still acquiring numerical abilities (in the Spanish educational system, numerical abilities are one of the goals of Primary Education, which takes place from 6 to 12 years old). Therefore, to avoid any effects of numerical competence and to make the task relatively easy and attractive for children, we decided to use a child-friendly visual scale with a reduced range of values, and without numbers. We did not allow intermediate units in this scale, since this would require children to manage and mentally operate with fractions of the unit (e.g., half a star; note that formal instruction about fractions is not introduced in Spanish schools at this age).

The experimenter verbally described the evaluation process, and how to use the scale as follows: “OK, this contestant has already finished. Now it is your turn to make your first important decision as judge: How good do you think the boy is at planting? Do you think he planted good, bad or so-so? Let’s give him stars depending on your judgment: If you consider that he performed perfectly, we can give him all the stars, if you consider that he performed very poorly we won’t give him any, and if he planted neither very poorly nor perfect, we can give him some stars: the better he performed the greater number of stars. How many of these stars do you want to give him?”

After rating the character’s performance, a new starting screen with “Contestant 2” written on the top was shown, announcing the second stage (see S1 Appendix for further details). In this new stage, participants were presented with another 24 training trials related to the other contestant. The procedure was exactly as that described for the first stage, except that a different scenario was used (different character, different layout, flowers instead of strawberries, etc.).

Once the second contestant had been rated, the trophy assignment screen (which featured the two characters and a trophy, see Fig 2I) was presented. At this point, the child was forced to choose between the two contestants, selecting who deserved the trophy.

Both contestants were poor farmers, as the contingency between planting and sprouting was set to zero for both, that is, p(Plants |Planting) = p(Plant |~Planting). However, the overall probability of the outcome, p(O), was high for one character, and low for the other: for the character in the High p(O) condition, the plants came out on 20 out of 24 trials, that is, p(O) = .83; whereas for the one in the Low p(O) condition, they came out on only 4 out of 24 trials, that is, p(O) = .17 (see Fig 1B for details).

Trials were randomly presented in two blocks of 12 trials each, with frequencies maintained proportional on each block and no visual or temporal gaps between them. The order in which the two p(O) conditions were presented and the scenarios assigned to each condition (character, plants, layout, contest background, gardening tools, etc.), were fully counterbalanced across participants.

### Results and discussion

As detailed previously, both characters were actually poor farmers but the plants grew with high probability for one character and with low probability for the other. Therefore, and if outcome probability actually biases children’s performance, they will assign a higher rating to the character in the High p(O) condition than to the character in the Low p(O) condition. Similarly, they will exhibit a preference for the former character when choosing the best farmer (trophy assignment).

**Fig 4:**
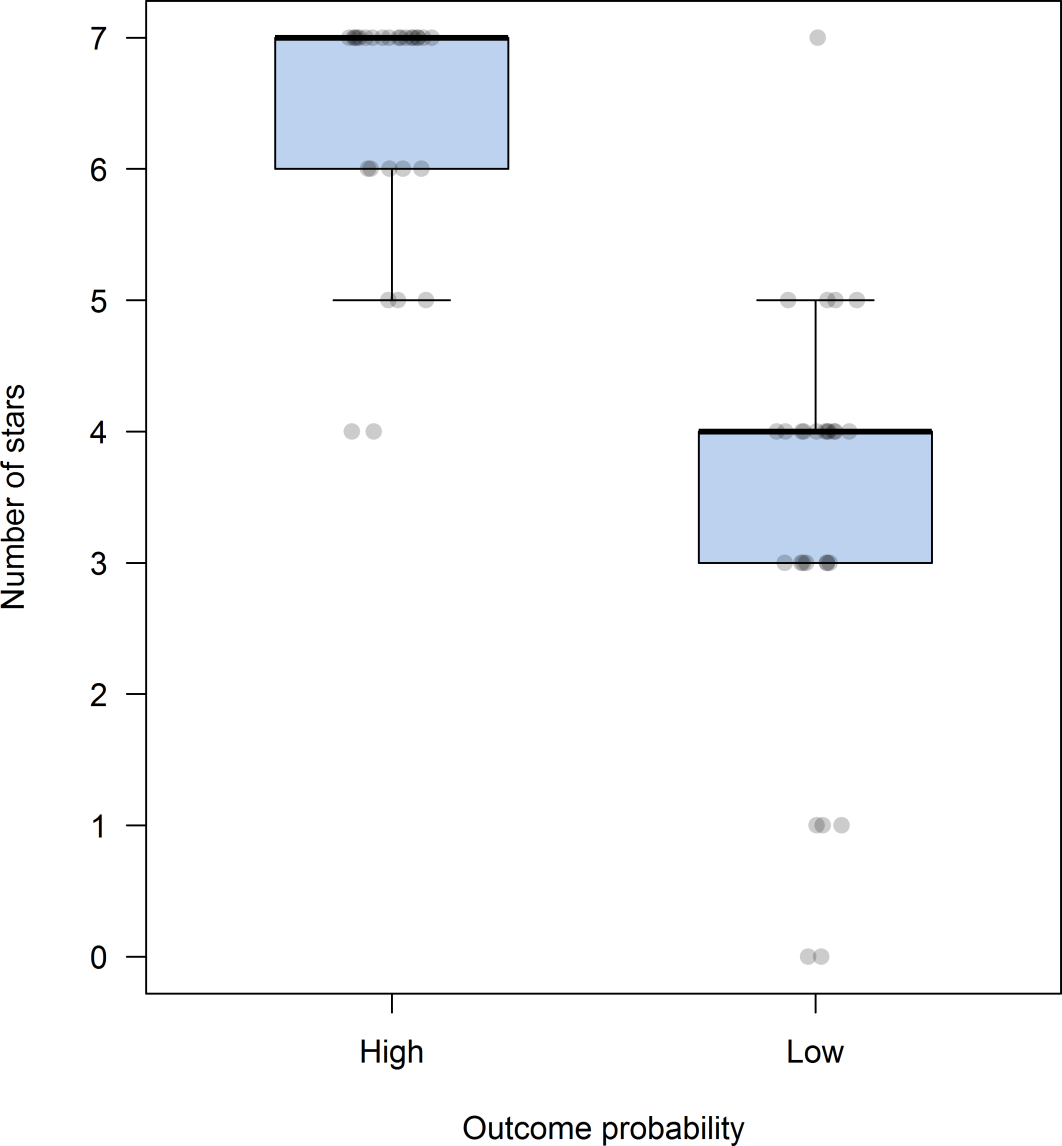
Box-plots showing the number of stars assigned to each contestant in Experiment 1. Boxes represent the middle 50% of the data (interquartile range, IQR) and whiskers the largest and lowest cases within 1.5 times the IQR. The dark line within each box represents the median. The dots represent the ratings of individual participants for each condition (jitter was added to each point to reduce overlapping).

Scores assigned to each contestant (i.e. number of stars assigned to the High and Low p(O) condition) are summarized in Fig 4. In both conditions, the data are skewed to the left. The median in the High p(O) condition matches the maximum value of the scale (7 stars) and, even if contingency was set to zero, all participants rated the character’s performance with at least 4 stars. Scores for the contestant in the Low p(O) condition were clearly lower than those in the High p(O) condition (*Mdn* = 4), Wilcoxon’s signed rank test *Z* = - 4.58, *p* < .001, with a large effect size (*r* = -0.61). All the relevant data are available as S1 Dataset.

Consistent with this result, the forced-choice response during the final evaluation showed that all participants selected the contestant in the High p(O) condition as the best farmer.

Given that the only aspect in which the contestants differed was the outcome probability, that is, the rate at which plants grew in each condition, these results are consistent with an outcome-density bias.

## Experiment 2

The results from the previous experiment suggest that children’s performance was biased by the outcome probability. Children systematically gave higher ratings to the character in the High p(O) condition, that is, the farmer that yielded a higher number of plants throughout the contest. However, both characters were actually poor farmers, since there was no contingency between seeding and the growing of the plants. It might be possible that the way in which the goal of the game is presented (a competition between both characters) may have caused the participants to focus on the characters rather than on the action of planting, leading children to believe that they have to give the highest score and trophy to the character who yielded a higher number of plants. In that case, and if participants are focused on the character, which appeared on all trials, it is possible for them to undervalue or even ignore the status of the actual cue (i.e. planting). Although cue status was stressed visually and verbally on each training trial, it is still possible that they ignored this information.

Experiment 2 was designed to control for this alternative interpretation. Participants were required to help the experimenter to find out if two products (powder and liquid) were actually useful to make plants grow. To achieve this goal, two characters would help them by going to the countryside to test each product (note that the characters now play a trivial role in the story). This slight change in the procedure allows for clearly distinguishing the cue status on each trial and avoids any potential confusion between the target cue and other contextual cues (i.e. characters) along the training phase. Additionally, all contextual cues will be removed on test to avoid contextual effects at this point. We expect to replicate the main findings from Experiment 1.

### Method

#### Ethics statement

The ethical review board of the University of Deusto examined and approved the procedure used in this experiment (Ref: ETK-12/14-15).

#### Participants

Nineteen children (12 boys) ranging from 6 to 7 years old (77 to 89 months, *M* = 82.32, *SD* = 3.92) were recruited from a public school in an industrial city, using a similar protocol to that described in Experiment 1.

#### Stimuli and apparatus

**Fig 5:**
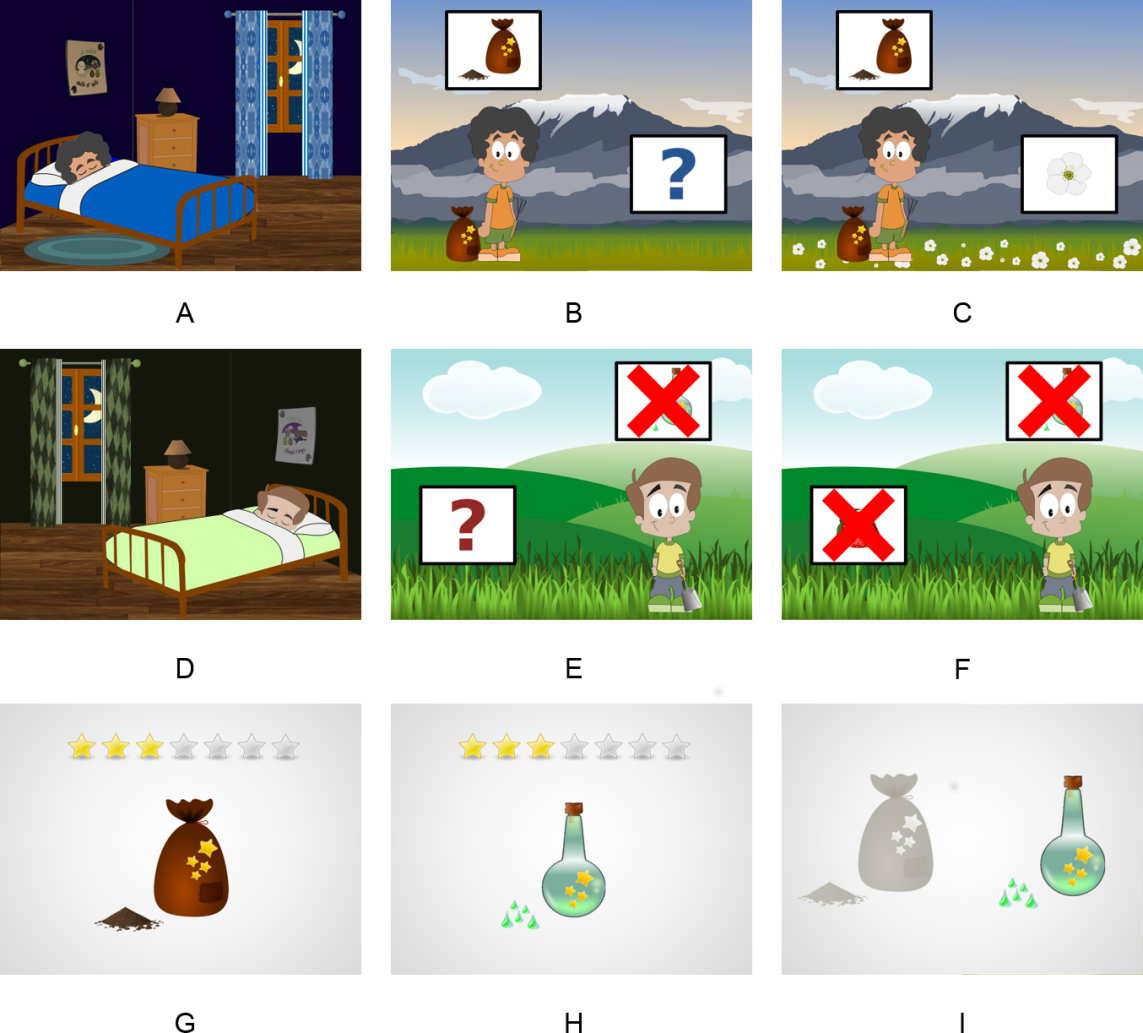
Example of the images used in Experiment 2. The first and second rows depict the training trial structure for each storyline. The panels correspond to: Inter-trial interval screens (panels A and D), Cue screens (panels B and E), and Outcome screens (panels C and F). Contestants carried products only on cue-present trials (cue-box placed on the upper side of the screen appeared crossed out otherwise). In the outcome screens, plants covered the ground on outcome-present trials (the outcome-box appeared crossed out otherwise). The third row depicts product evaluation screens (panels G and H), and the final evaluation screen (panel I). The figures were adapted from images retrieved from https://openclipart.org under a CC BY license, with permission from https://openclipart.org, original copyright CC0.

The stimuli used in Experiment 1 were slightly modified to fit the new cover story, and to avoid any confusion about the cue under evaluation. Thus, instead of the gardening tools used in Experiment 1, the cue was depicted either as a bag with a small amount of powder on the right side, or a blue bottle with small drops of liquid, also on the right side. Individual evaluation screens were also modified to depict only the product under assessment. Similarly, only the two products were portrayed on the final forced-choice screen (see Fig 5 for details). All other details were identical to those described for Experiment 1.

#### Procedure and design

The procedure was almost identical to that described in Experiment 1 (see Fig 3), with minor changes mainly derived from variations in the cover story. The program is available at the Open Science Framework [79].

The children were asked not to judge the characters’ performance, but the reliability of two planting products. To achieve this goal, they were informed that two characters would help them by going to the countryside to test each product (see S2 Appendix for further details).

The participants were then presented with 24 trials related to the first product. The trial structure was the same as that employed in Experiment 1. Each trial began with an inter-trial interval with the character sleeping (see Fig 4A and 4D). The child was asked to wake him up in the same way as described for the previous experiment, and the cue screen was subsequently presented. The white box presented in the upper side of the image may contain the product either crossed out or not (see Fig 4B and 4E). Instructions regarding the cue status (present or absent) were also similar to those previously used: “Look, he is [is not] carrying the liquid [powder]”.

Participants were asked to move into the outcome screen by clicking on the question mark, as in Experiment 1. Subsequently, a plant (either crossed out or not) appeared instead of the question mark, and the ground was covered with plants (on outcome-present trials) or remained unchanged (on outcome-absent trials), as shown in Fig 5C and 5F. Outcome presence or absence was also stressed on each trial: “Look, flowers [strawberries] have [have not] grown, let’s see what will happen the next day”.

After 24 training trials, the participants were asked to evaluate the product by assigning stars from 0 (not effective at all) to 7 (very effective). The experimenter described the evaluation process, and how to use the scale as follows: “OK, we have finished testing the liquid [powder]. Now it is very important that you help me to decide if it works fine, bad or so-so. Let’s give it stars, depending on your opinion. If you consider that the product works perfectly we can give it all the stars, if you consider that it works very badly we won’t give it any, and if it works either very badly or not perfectly we can give it some stars: the better it works the greater number of stars. How many stars do you want to give it?” Note that characters are not even mentioned at this point. The picture depicted on the evaluation screen presented only the product under evaluation on a grey background that was not presented during the training phase, making it unlikely that participants misinterpret the task as involving direct evaluation of the characters or of any other contextual cue (see Fig 4G and 4H).

Once the number of stars had been selected and the response confirmed by clicking on the product, the second stage began (see S2 Appendix for additional details). In this new stage participants were presented with a further 24 training trials related to the other product, following the same procedure already described. After assessing the second product, a final screen with the two products was presented (see Fig 5I), and the child was asked to choose which one worked better, answering a forced-choice question as in Experiment 1.

#### Results and discussion

**Fig 6:**
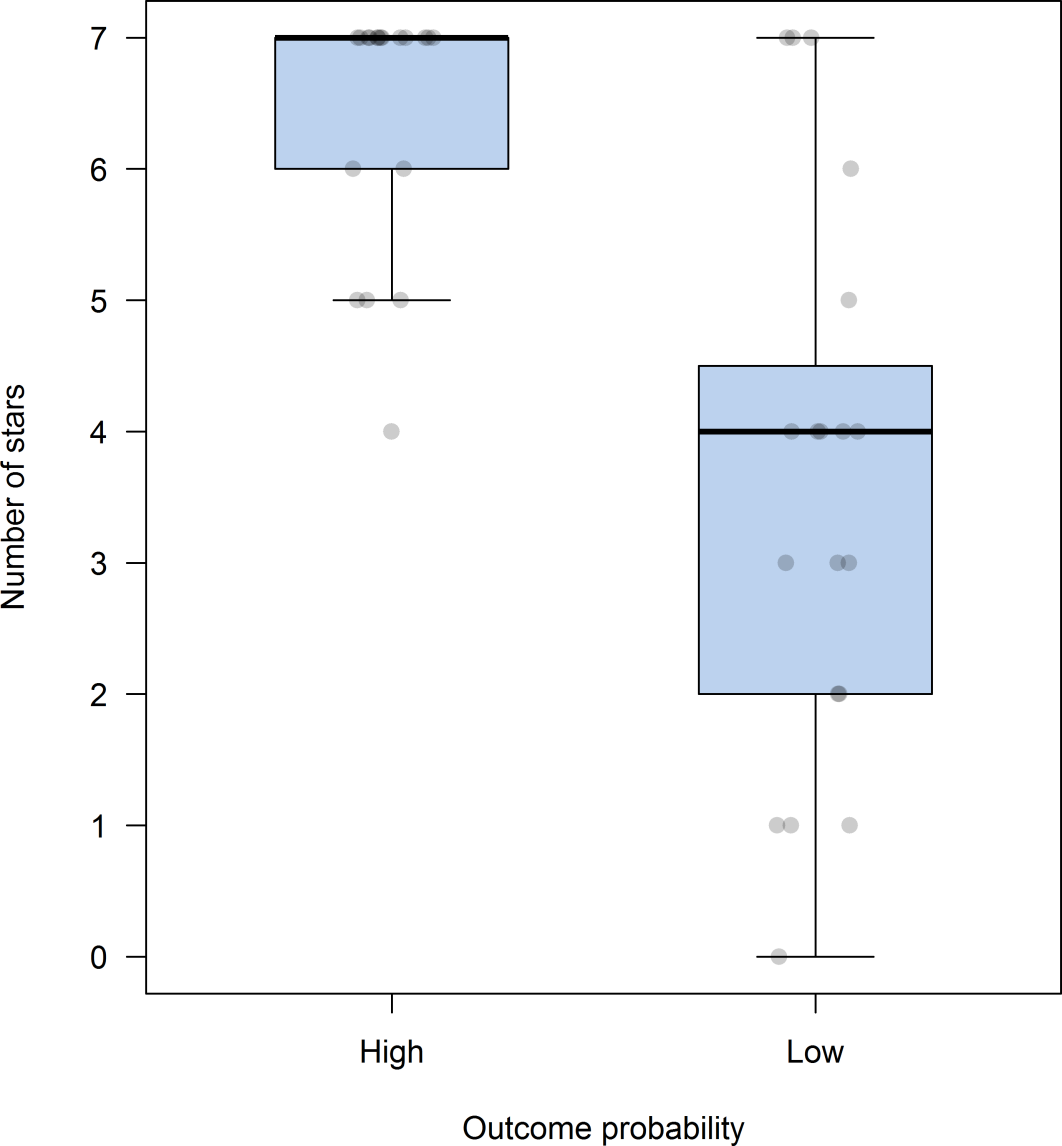
Box-plots showing the number of stars assigned to each contestant in Experiment 1. Boxes represent the middle 50% of the data (interquartile range, IQR) and whiskers the largest and lowest cases within 1.5 times the IQR. The dark line within each box represents the median. The dots represent the ratings of individual participants in each condition (jitter was added to each point to reduce overlapping).

Ratings assigned to each product (i.e. number of stars) are depicted in Fig 6. Data distribution on the High p(O) condition was clearly skewed to the left, with most responses concentrated between five and seven stars, and just one evaluation below five stars (*Mdn* = 7). In fact, 68.4% of participants selected the highest value of the scale when assessing this product. This pattern of responding clearly differs from that in the Low p(O) condition, Wilcoxon’s signed rank test *Z* = -3.48, *p* = .001, with a large effect size (*r* = -0.56). Participants were more conservative in this condition, and their responses were distributed throughout the whole scale (*Mdn* = 4). The results of the forced-choice question (i.e. the question of which product was best) were convergent with those yielded from the above-mentioned ratings, and 95% of participants chose the product in the High p(O) condition as the one which worked better. All the relevant data are available as S1 Dataset.

Both measures (product ratings and final forced-choice response) revealed a biased perception of the products' efficacy. Although the products were equally inefficient—since the tendency for the plants to grow was non-contingent with their use—the results showed that the perceived effectiveness of the products was driven by the outcome probability, and the products were judged as more effective when the plants bloomed frequently than when they did not.

These results mimic those previously reported in Experiment 1, except that on this occasion the task was presented from the outset as an assessment of a product’s effectiveness rather than as a competition between characters. In particular, the characters (or other any contextual elements) were not significant features of the storyline, and their role was not emphasized or enhanced in this procedure. Thus, both during training and during test, the children were provided with an unequivocal cue that did not run the risk of being masked or confounded with others, as may be argued for Experiment 1, ensuring that participants were actually rating the effectiveness of the cue.

## General discussion

Causal learning is a significant tool for adapting to the environment. Humans are usually quite accurate in inferring causality from experience, but there are some factors that can bias their judgments of causality [12,78]. One of these factors is the probability of the outcome, which typically leads to the *outcome-density bias* [14,15,17,75,76]. This bias has not yet been reported in children, and we have proposed a number of reasons to suspect that children might differ from adults in their performance.

The two experiments reported in this paper explore the outcome-density bias in a group of children as young as 6 years, extending previous research to childhood, a period in which certain cognitive abilities that are crucial for causal inference are still developing. The results have shown that when estimating the relation between two unrelated events, children, like adults, may be influenced and biased by the outcome probability.

In Experiment 1, children were required to act as judges of a competition between the two characters. Their performance was clearly biased by the outcome probability, rating higher the character on the High p(O) condition and considering this character to be worthy of the trophy. Although children were specifically asked to judge how well each character planted, to a certain extent the way in which this goal was presented in Experiment 1 may have encouraged participants to focus on characters while ignoring the actual cause under evaluation (i.e. planting). If so, and given that characters are always present (either planting or not), all trials can be considered as cue-present trials, and the overall probability of the outcome is the only information available to guide the children in their judgments. In this case, the children may have relied on the number of plants grown in the presence of each character and not on the characters’ abilities to plant, eventually choosing the High p(O) character as the best one simply because, regardless of the cause, he yielded more plants than the other. In our view, this explanation seems questionable given that the verbal instructions and visual information served to emphasize the status of the cue on each trial. Nevertheless, we conducted a second experiment to rule out this possibility.

In Experiment 2 we changed the cover story, and eliminated any visual cues that may have led to confusion about the goal of the participant on test. In this way, the procedure was rendered more similar to those commonly used in adults, since it entails evaluating the effectiveness of a product rather than the more abstract evaluation of an action (i.e. planting). The results replicated those of Experiment 1: as expected, the judgments were still biased by outcome probability. To our knowledge, this is the first time that the outcome-density bias has been reported in children, though some aspects of the results deserve further discussion. First, and although the two participating schools were gender-mixed and no gender selection criteria were applied, the distribution of this variable was different between the two experiments, with a higher number of girls in Experiment 1 and a higher number of boys in Experiment 2. Previous research suggests that gender could be a relevant factor in tasks involving self-attribution, that is, in active procedures in which the participant’s actions are the potential cause under evaluation. In those situations, a greater illusion of control has been reported in females than in males [3,80]. However, gender effects are not usually reported in passive/observational procedures like the one used in this research. Therefore, we did not expect any influence of gender in our results, because the procedure did not involve evaluations about self-performance, and, most importantly, because the outcome-density effect was measured within subjects. Accordingly, despite the different gender distribution in the samples, both experiments showed similar results: an outcome-density bias.

An additional aspect of the data that is worth noting is that both experiments showed a left-skewed distribution of the judgments, and a consistent ceiling effect in the High p(O) condition. In contrast, previous research on the outcome-density effect in adults has reported much less extreme distributions of responses. For instance, Blanco, Vadillo and Matute [75] evaluated the outcome-density bias in a situation in which the probability of the outcome was either .80 or .20 (two probabilities that are very similar to the ones used for the current Experiments 1 and 2, i.e., .83 and .17). Although the authors reported overestimations of the causal relation between the two unrelated events under evaluation, causal ratings were not as high as the ones reported here, even though Blanco et al. [75] included an additional manipulation that was shown to boost the outcome-density effect in the High p(O) condition (a similar situation applies to [78]).

The generally high ratings obtained in our two experiments with children may be explained either from a developmental perspective (considering these extreme responses as a developmental feature of the outcome-density bias) or as a methodological issue (consequence of necessary deviations from the standard procedure). The former option can be related to children’s preference for extreme responses when rating abstract concepts or subjective and unobservable states. Previous research has already highlighted the skewed use of scales by young school-age children [81], and although this tendency has not been specifically explored within the domain of causal reasoning, ceiling effects found in this research may be reflecting young children’s preference to endorse responses at the extreme end of a scale. Although this could explain the High p(O) condition, this explanation cannot account for the judgments in the Low p(O) condition, since a floor effect should be expected in this case.

The other possible explanation for the unusually high ratings in our two experiments is related to certain deviations from the standard procedure used in adults. As explained above, developmental characteristics of children forced us to modify the standard procedure in certain ways; among other changes, we used an adult-supervised procedure with a new cover story, a reduced number of trials, and a different measure of contingency learning (rating characters/products with 0–7 stars rather than with a 0–100 numerical scale). These variations may have contributed towards producing extreme judgments in different ways. For example, we have already noted that the relative frequency of each type of trial is similar to that of several adult experiments [75,78]. However, the absolute number of trials (i.e. 24 trials on each condition) is below the typical range used in adult research, which is usually around 40–50 trials [12]. In fact, associative accounts of the outcome-density effect, such as Rescorla and Wagner’s [82], predict an overestimation of contingency during the initial stages of learning or, in other words, higher causal judgments after a short training phase than after a long phase [83]. That is, the reduced number of trials may increase the bias and thus be responsible for the high ratings reported in both experiments.

While differences between the High and Low p(O) condition can be detected with this procedure, methodological differences discourage direct comparison between children and adult performance. Nevertheless, and despite the mentioned limitations, the experiments reported here provide evidence to suggest that the outcome-density effect can bias causal judgments at early ages, and they offer an adapted procedure that will allow for a further characterization of the outcome-density bias during childhood. Future research should evaluate the factors underlying the discrepancies with adult research described here, disentangling the contributions from developmental and methodological factors, and providing a deeper understanding of the effect throughout life.

## Supporting information

S1 Appendix Additional instructions and pictures of Experiment 1. (DOCX)

S2 Appendix Additional instructions and pictures of Experiment 2. (DOCX)

S1 Dataset Datasets of Experiment 1 and 2. (XLSX)
