# Supplementary material for: Causal illusions in children when the outcome is frequent
Source: PLoS One. 2017 Sep 12;12(9):e0184707. doi: 10.1371/journal.pone.0184707 (PMC5595306; doi:10.1371/journal.pone.0184707)
Supplement: S1 Appendix — (DOCX) [file pone.0184707.s001.docx]

**S1 Appendix**

**Additional instructions and pictures of Experiment 1.**

Additional instructions are translated from Spanish:

[Initial Instruction] “We are about to see how these two boys take part in a strawberries and flowers seedling contest. This boy will always sow flowers like this one, and this other boy will always sow strawberries like this one. Sometimes they will seed properly and the plants will grow, but on other occasions they will do it wrong and plants will not grow. Be aware that some days they will go to the contest but they will not be required to plant (you will see that they do not carry the tools because they are not required to sow); even in those cases, it is possible that strawberries and flowers grow by themselves. Have you ever seen flowers and strawberries in the countryside? Nobody sows them, but they grow by themselves.

You are going to be the judge of this contest! Pay careful attention to what is happening with each contestant so that you can tell me if they sow well, bad, or so-so. At the end, you will have to decide who is the best farmer to give him a trophy. Is that okay with you? Shall we start? Let’s see who does it better!”

[First training stage instructions] “Do you know how to use the mouse? Ok, then you are going to use the mouse. Click here to see what happens with the first contender!”

[Second training stage instructions] “Now let’s see what happens when the second contender, pay careful attention to see if he does it better because you will have to decide who is the best farmer to give him a trophy”

**
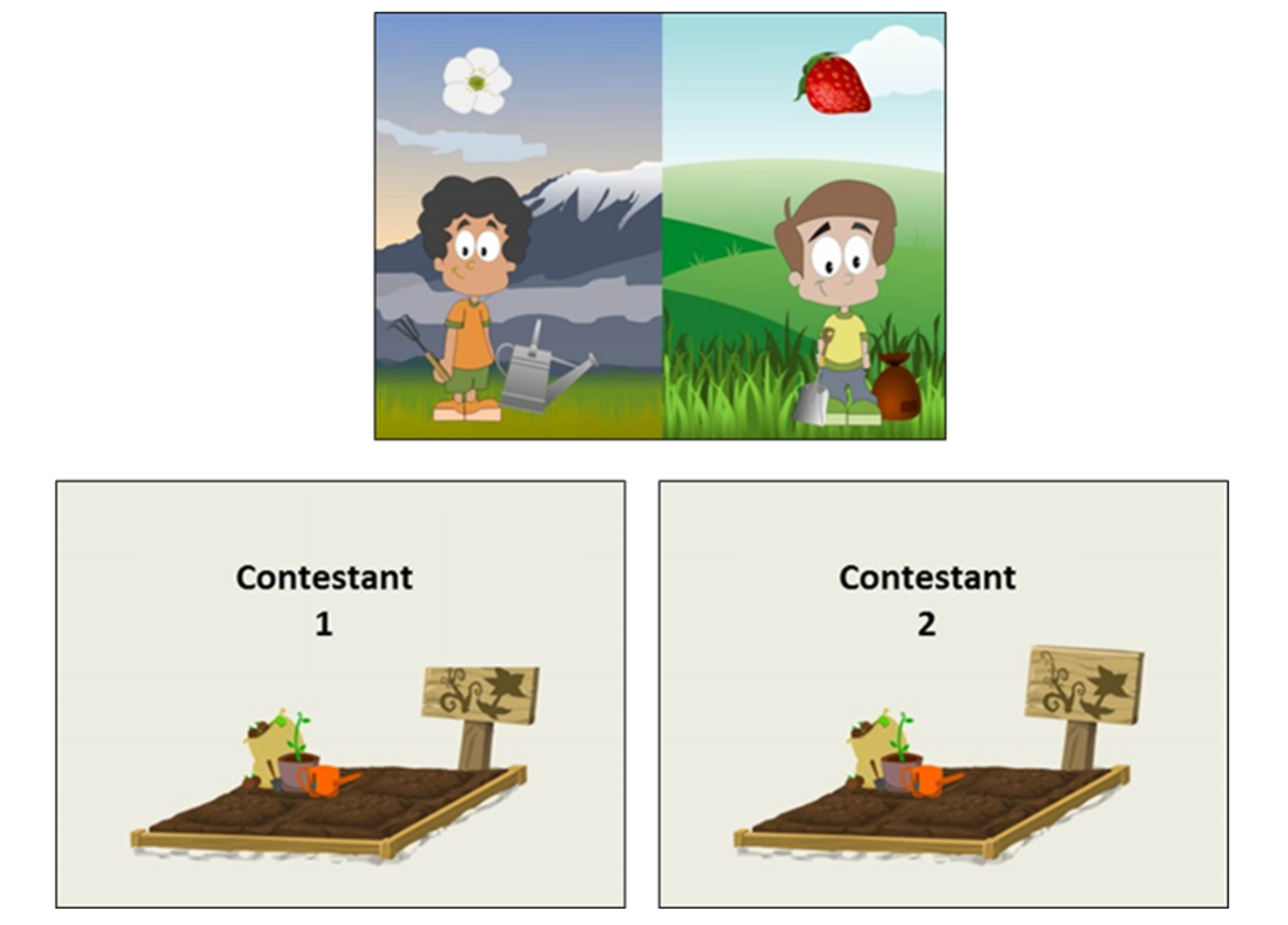
**

**Fig A. Pictures presented throughout the initial instructions of Experiment 1.** The first row depicts the picture presented during the initial instructions, and the second row the pictures used to announce the first (left) and second stage (right). Figures adapted from images retrieved from https://openclipart.org under a CC BY license, with permission from https://openclipart.org, original copyright CC0.
