# Supplementary material for: Causal illusions in children when the outcome is frequent
Source: PLoS One. 2017 Sep 12;12(9):e0184707. doi: 10.1371/journal.pone.0184707 (PMC5595306; doi:10.1371/journal.pone.0184707)
Supplement: S2 Appendix — (DOCX) [file pone.0184707.s002.docx]

**S2 Appendix**

**Additional instructions and pictures of Experiment 2.**

Additional instructions are translated from Spanish:

[Initial instruction, Screen 1] “I have this powder bag and this bottle with liquid. The powder might be useful for making flowers bloom and the liquid for making strawberries grow. However, they must be tested and I need somebody to help me. Do you want to help me?

Great! I am going to explain to you what to do”

[Initial instruction, Screen 2] “These two boys are going to the countryside to test the powder and the liquid so that you can see how they work. This one is going to help you with the powder and this one with the liquid. The problem is that both products are difficult to find and the boys will not be able to use them every day: some days the will used the products for planting, and on other days they will have to plant without them. What is your task?

You have to pay attention in order to help me decide if the products are useful to make the plants grow. Take note of what happens each day and at the end tell me which product works better. Is that okay with you? Shall we start?”

[First training stage instructions] “Do you know how to use the mouse? Ok, then you are going to use the mouse. Click here to see what happens with the powder [liquid]!”

[Second training stage instructions] “Now let’s see what happens when the liquid [powder] is used, pay careful attention”


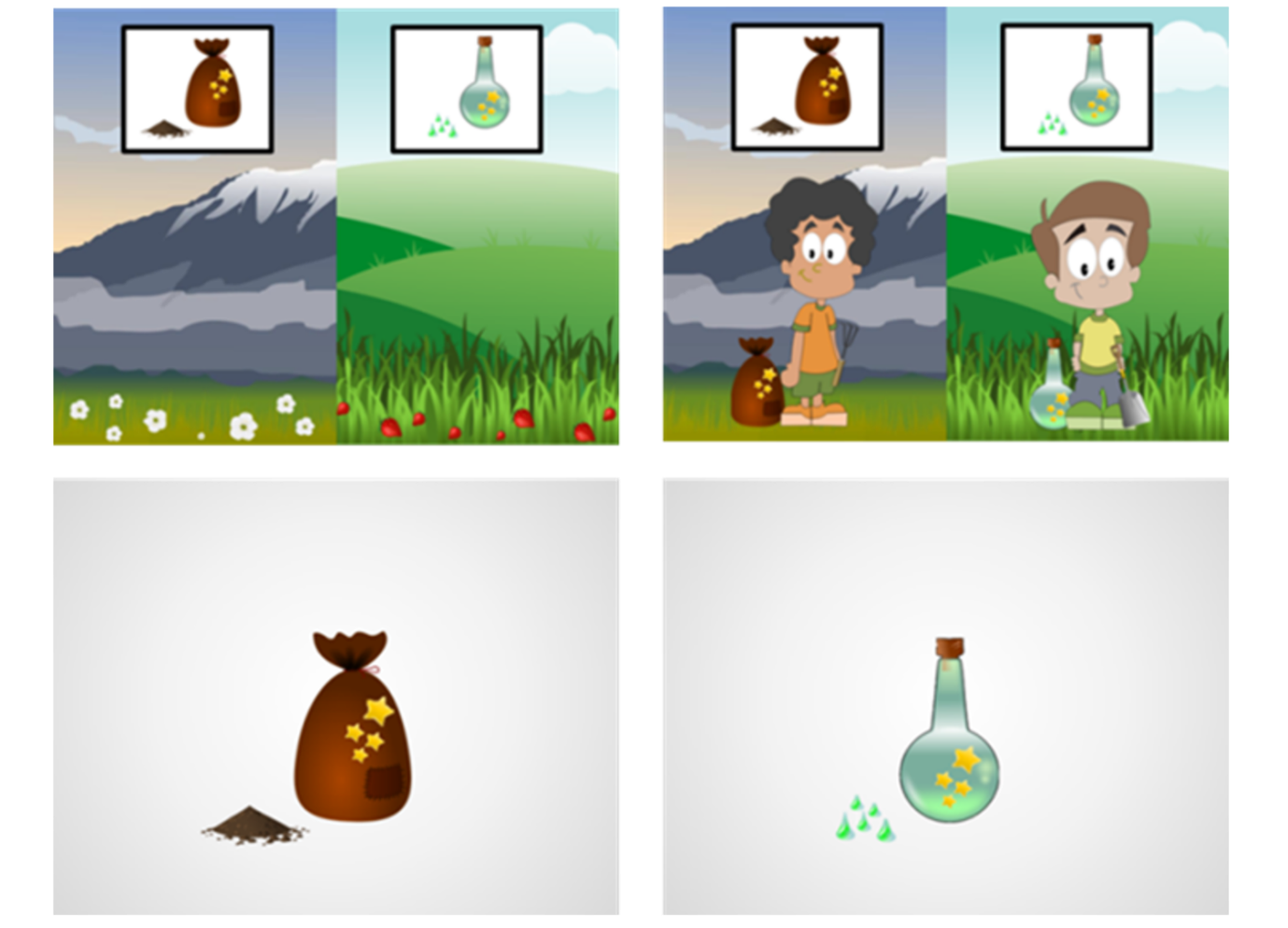


**Fig A. Pictures presented throughout the initial instructions of Experiment 2.** The first row depicts the pictures presented during the initial instructions, and the second row the pictures used to announce the first and second stage. Figures adapted from images retrieved from https://openclipart.org under a CC BY license, with permission from https://openclipart.org, original copyright CC0.
